# Transcriptome Analysis of the Midgut of the Chinese Oak Silkworm *Antheraea pernyi* Infected with *Antheraea pernyi* Nucleopolyhedrovirus

**DOI:** 10.1371/journal.pone.0165959

**Published:** 2016-11-07

**Authors:** Xi-Sheng Li, Guo-Bao Wang, Ying Sun, Wei Liu, Ying-Zi He, Feng-Cheng Wang, Yi-Ren Jiang, Li Qin

**Affiliations:** 1 College of Plant Protection, Shenyang Agricultural University, Shenyang, 110866, China; 2 College of Bioscience and Biotechnology, Liaoning Engineering & Technology Research Center for Insect Resources, Shenyang Agricultural University, Shenyang, 110866, China; 3 Sericultural Research Institute of Liaoning Province, Fengcheng, 118100, China; USDA Agricultural Research Service, UNITED STATES

## Abstract

The *Antheraea pernyi* nucleopolyhedrovirus (ApNPV) is an exclusive pathogen of *A*. *pernyi*. The intense interactions between ApNPV and *A*. *pernyi* cause a series of physiological and pathological changes to *A*. *pernyi*. However, no detailed report exists regarding the molecular mechanisms underlying the interactions between ApNPV and *A*. *pernyi*. In this study, four cDNA libraries of the *A*. *pernyi* midgut, including two ApNPV-infected groups and two control groups, were constructed for transcriptomic analysis to provide new clues regarding the molecular mechanisms that underlie these interactions. The transcriptome of the *A*. *pernyi* midgut was de novo assembled using the Trinity platform because of the lack of a genome resource for *A*. *pernyi*. Compared with the controls, a total of 5,172 differentially expressed genes (DEGs) were identified, including 2,183 up-regulated and 2,989 down-regulated candidates, of which 2,965 and 911 DEGs were classified into different GO categories and KEGG pathways, respectively. The DEGs involved in *A*. *pernyi* innate immunity were classified into several categories, including heat-shock proteins, apoptosis-related proteins, serpins, serine proteases and cytochrome P450s. Our results suggested that these genes were related to the immune response of the *A*. *pernyi* midgut to ApNPV infection via their essential roles in regulating a variety of physiological processes. Our results may serve as a basis for future research not only on the molecular mechanisms of ApNPV invasion but also on the anti-ApNPV mechanism of *A*. *pernyi*.

## Introduction

*Antheraea pernyi* is not only an important economic insect but also a rising model organism because of a variety of advantages, such as its ease of rearing and experimental manipulation compared with other Lepidoptera insects. However, *A*. *pernyi* nucleopolyhedrovirus (ApNPV), a major viral pathogen for *A*. *pernyi*, causes enormous damage to *A*. *pernyi* and also the sericulture industry. ApNPV belongs to the NPV subfamily, which displays two types of phenotypes with the same genetic resource during its infection cycle, including budded virus (BV) and occlusion-derived virus (ODV). ODV has high infectivity to the intestinal epithelial cells of the host. The ODV particles invade the peritrophic membrane of the midgut by oral infection, and drop off their capsules through an interaction between the microvilli of the intestinal epithelial cells and the capsule. The infection is initiated with the nucleocapsid of ODV releasing into the intestinal epithelial cells [1]. Then, the host initiates resistance strategies to block viral infection, such as through the immune system, apoptosis, and RNA interference [2]. The infected *A*. *pernyi* larva exhibits several symptoms in the late stage of infection, such as no feeding, various sizes of circular pus blotches on the epidermis, abnormal behavior, and finally death. However, no detailed report exists regarding the molecular mechanisms underlying these interactions between ApNPV and *A*. *pernyi*.

Changes in host gene expression in response to viral infection are of great interest. Research has been focused on gene expression changes in the midgut in insects because of its important role in resisting pathogen invasion and proliferation as the first line of innate immunity in the host [3]. In the midgut of *Aedes aegypti*, silencing of 5G1, which belongs to the midgut serine protease family, or the soybean trypsin inhibitor significantly increased midgut infection rates of Dengue virus 2 (DENV-2), suggesting that some midgut serine proteases may limit DENV-2 infectivity of *A*. *aegypti* [4]. Smartt *et al*. (2009) identified 26 differentially expressed cDNA clones in the midgut tissue of *Culex pipiens quinquefasciatus* after exposure to West Nile virus (WNV) [5]. Using Digital Gene Expression analysis, Gao *et al*. (2014) identified 752 differentially expressed genes, including 649 up-regulated and 103 down-regulated genes, in the *B*. *mori* cytoplasmic polyhedrosis virus (BmCPV) infected midguts of 4008 silkworm strains [6]. Kolliopoulou *et al*. (2015) obtained 308 differentially expressed genes in *Bombyx mori* larval midgut infected with BmCPV through transcriptome analysis, some of which are involved in physical barriers and immune responses [7].

RNA-sequencing (RNA-Seq) based on deep sequencing technologies is a powerful and high-throughput method for transcriptome analysis. RNA-Seq has been widely used to investigate the molecular mechanisms of the interactions between pathogens and their insect hosts [8–11]. In this study, RNA-Seq was applied to polyadenylate-enriched mRNAs from *A*. *pernyi* midguts to better understand the complexity of the molecular mechanisms underlying the interactions between ApNPV and the *A*. *pernyi* midgut at the transcriptional level. We first assembled the transcriptome sequences and then identified differentially expressed genes (DEGs), including both up-regulated and down-regulated genes in ApNPV-infected midguts compared with controls. We also screened immune-related genes from the DEGs via bioinformatic analysis. The results of this study provide useful information for further research on the molecular mechanisms underlying the ApNPV-induced nuclear polydedrosis virus disease of *A*. *pernyi*.

## Materials and Methods

### Sample preparation

The *A*. *pernyi* strain Jiaolan stored in our laboratory was used in this study. The larvae were maintained in a rearing chamber at 23 ± 2°C with 70 ± 5% relative humidity and fed with fresh leaves of *Quercus mongolic* collected from the research base of Shenyang Agricultural Uinversity (Shenyang city, 41.8 N°, 123.4°E). After molting twice, the third instar *A*. *pernyi* larvae were separated into two groups randomly. The experimental group marked as Ap_NPV was fed with the leaves added with 4.05×10^6^ polyhedra/mL for three days and then fed with fresh leaves. For the non-infected controls, the same volume of 0.9% physiological saline was mixed in the feed for the *A*. *pernyi* larvae, and the rearing conditions were identical to those of the ApNPV-infected groups. Each group contained twenty five *A*. *pernyi* larvae. The midguts were dissected from the two groups when polyhedra could be observed in the hemocytes of ApNPV-infected *A*. *pernyi* larvae under a microscope. All experiments were performed with two independent biological replicates.

### Total RNA extraction

Total RNA was extracted using TRIzol^®^ Reagent (Invitrogen) according to the manufacturer’s protocol. The RNA was quantified by measuring the absorbance at 260 nm using a NanoVue UV-Vis spectrophotometer (Bio-Science). RNA purity was checked using a NanoPhotometer^®^ spectrophotometer (IMPLEN, CA, USA). RNA integrity was assessed using the RNA Nano 6000 Assay Kit of the Agilent Bioanalyzer 2100 system (Agilent Technologies, CA, USA).

### cDNA library construction and Illumina RNA-Seq

Library construction and RNA-Seq were performed by the Novogene Experimental Department (Beijing, China). Briefly, a total of 3 μg RNA per sample was used as input material for the RNA sample preparations. Sequencing libraries were generated using the NEBNext^®^ Ultra™ RNA Library Prep Kit for Illumina^®^ (NEB, USA) following manufacturer’s instructions. The mRNA was purified using poly-T oligo-attached magnetic beads. Fragmentation was performed using divalent cations under elevated temperature in 5×NEB Next First Strand Synthesis Reaction Buffer. First strand cDNA was synthesized using a random hexamer primer and M-MuLV Reverse Transcriptase (RNase H^-^). Second strand cDNA synthesis was subsequently performed using DNA Polymerase I and RNase H. The remaining overhangs were converted into blunt ends via exonuclease/polymerase activities. After adenylation of the 3’ends of the DNA fragments, NEBNext Adaptors with hairpin loop structures were ligated to prepare for hybridization. To select cDNA fragments of preferentially 150–200 bp in length, the library fragments were purified with the AMPure XP system (Beckman Coulter, Beverly, USA). Then, 3 μL USER Enzyme (NEB, USA) was added to size-selected, adaptor-ligated cDNA at 37°C for 15 min followed by 5 min at 95°C before PCR. PCR was performed with Phusion High-Fidelity DNA polymerase, Universal PCR primers and an Index (X) Primer. Finally, PCR products were purified (AMPure XP system) and library qualities assessed on an Agilent Bioanalyzer 2100 system. Clustering of the index-coded samples was performed on a cBot Cluster Generation System using the TruSeq PE Cluster Kit v3-cBot-HS (Illumina) according to the manufacturer’s instructions. After cluster generation, the library preparations were sequenced on an Illumina Hiseq 2500 platform and paired-end reads were generated.

### De novo transcriptome assembly

Reads with only adaptors, > 5% unknown nucleotides, or low quality reads were first filtered. The quality reads were assembled into unigenes using the Trinity software [12]. The gene expression levels were estimated by RSEM [13] for each sample. Firstly, clean data were mapped back onto the assembled transcriptome. Then, read count for each gene was obtained from the mapping results. The FPKM measure (Fragments per Kilobase per Millions base pairs sequenced) [14, 15] was used to calculate unigene expression. The transcripts spliced by Trinity were used as reference sequences (ref), and the clean reads of each sample were mapped onto the ref using the RSEM software. The read count of each unigene in each sample was converted to FPKM to obtain gene expression. A FPKM threshold of > 0.1 was applied to ensure that the genes were expressed in both of the two groups. Differential expression analysis was performed using the DESeq R package (1.10.1). The resulting P values were adjusted using the Benjamini and Hochberg’s approach for controlling the false discovery rate. Genes with an adjusted P-value < 0.05 found by DESeq were assigned as differentially expressed.

### Bioinformatic analysis

The obtained unigenes were aligned to a series of databases using BLASTx (E-value ≤ 10^−5^), including the NCBI non-redundant (Nr), Swiss-Prot, Trembl, Kyoto Encyclopedia of Genes and Genomes (KEGG) (http://www.genome.jp/kegg/kegg2.html) and Gene Ontology (GO) (http://wego.genomics.org.cn/cgi-bin/wego/index.pl) databases and the corresponding annotation results were extracted.

### Validation of data reliability by quantitative RT-PCR

Quantitative RT-PCR (qRT-PCR) was used to confirm the expression profiles of genes that were identified from the Illumina sequencing analysis. Eight DEGs, including 5 down-regulated and 3 up-regulated genes, were selected for qRT-PCR. The gene-specific primers were designed using the predicted CDSs as reference sequences. The details of the primers are provided in S1 Table. qRT-PCR was performed on a LightCycler 480 Real-time Detection System (Roche Diagnostics). SYBR^®^Premix Ex TaqTMII (Tli RHaseH Plus) was mixed with 1 ng of template cDNA. The reaction was performed in a total volume of 10 μL, containing 5 μL of 2×SYBR Premix Ex Taq^TM^ (TaKaRa), 1 μL of diluted cDNA mix, 0.4 μL of each primer (10 mM) and 3.2 μL of Milli-Q water. Cycling conditions were as follows: 30 s at 95°C, 5 s at 95°C, followed by 40 cycles with denaturation for 20 s at 60°C and annealing/elongation for 15 s at 65°C. Melting curves were generated after each run to confirm a single PCR product. All reactions were performed in triplicate. The housekeeping gene actin3 was used as the endogenous control. After the PCR program, data were analyzed with Light Cycler 480 software (Applied Biosystems). The comparative CT method (2^-ΔΔCT^ method) [16] was used to analyze the expression levels of different genes.

### Ethics Statement

The leaves of *Quercus mongolic* were collected from the research base of Shenyang Agricultural University. We obtained the permission from Shenyang Agricultural University.

## Results

### Transcriptome analysis of larval midgut samples

**Fig 1:**
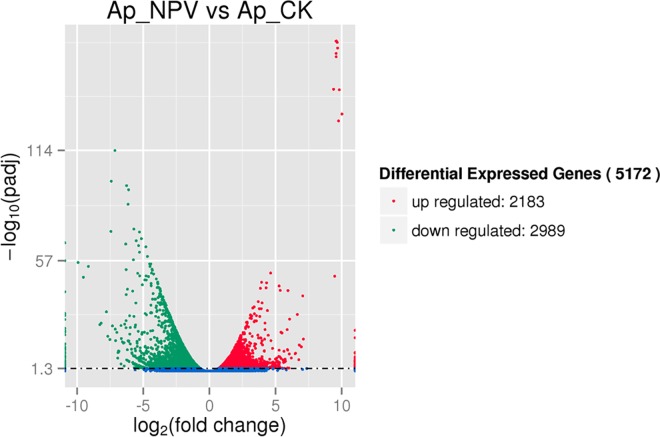
Volcano plot of the DEGs. The horizontal ordinate represents the fold change of gene expression in the different experimental groups, and the vertical ordinate represents the statistical significance of the change of gene expression. Each point in the plot represents each gene, and the red and green points represent the significant up- and down-regulated genes.

To obtain a global view of the transcriptome related to the response of the *A*.*pernyi* larvae midgut to ApNPV infection, we constructed four cDNA libraries, numbered correspondingly as Ap_CK1/Ap_NPV1 and Ap_CK2/Ap_NPV2, in which Ap_NPV refers to the tested samples and Ap_CK the control ones with two replicates for each group. The four cDNA libraries (Ap_CK1, Ap_CK2, Ap_NPV1 and Ap_NPV2) produced 71,989,178, 57,985,902, 70,871,812 and 62,299,960 raw 150 bp paired-end reads (We submitted these raw reads to NCBI Sequence Read Archive, and obtained their NCBI accession numbers: SRR2919240, SRR2919241, SRR2919242 and SRR2919243, respectively) with GC percentages of 45.59%, 45.73%, 44.32% and 43.87%, respectively. After removal of adapter sequences, low quality reads and N-containing reads, 61,595,738 (85.56%), 49,324,114 (85.06%), 60,792,564 (85.78%) and 52,512,994 (84.29%) clean reads were obtained in the four cDNA libraries, respectively (Table 1). We identified 5,172 differentially expressed genes (DEGs) from the ApNPV-infected groups compared with the controls, including 2,183 up-regulated (S2 Table) and 2,989 down-regulated genes (S2 Table). The distribution of the DEGs is shown in Fig 1.

**Table 1:** Statistical analysis of the transcriptome sequence data.

Sample	Raw Reads	Clean Reads	Error (%)	Q20 (%)	Q30 (%)	GC Content (%)
Ap_CK1	71989178	61595738	0.03	97.96	93.23	45.59
Ap_CK2	57985902	49324114	0.03	97.96	93.22	45.73
Ap_NP1	70871812	60792564	0.03	97.99	93.42	44.32
Ap_NP2	62299960	52512994	0.03	97.98	93.4	43.87

### Analysis of the pearson correlation between different samples

**Fig 2:**
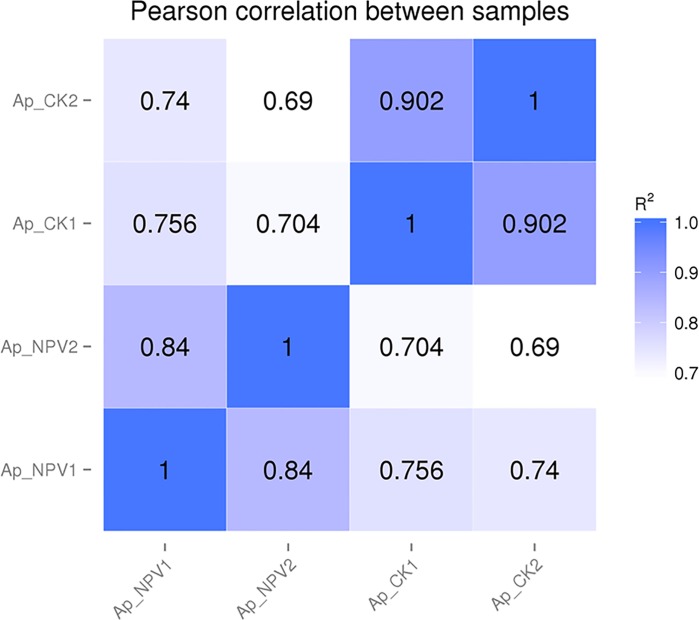
Pearson correlation between the samples in the Ap_CK and Ap_NPV groups.

The pearson correlation between different samples is an important parameter to test the reliability of the experiment and whether the sample selection is reasonable. In this study, we carried out two independent technology replications in each group. The pearson correlation between the two control samples was 0.902 with that was 0.84 between the two ApNPV infected samples, showing the high repeatability. The control and ApNPV infected samples showed low pearson correlation with each other, indicating the significant difference between them (Fig 2).

### Gene ontology (GO) analysis of the differentially expressed genes

**Fig 3:**
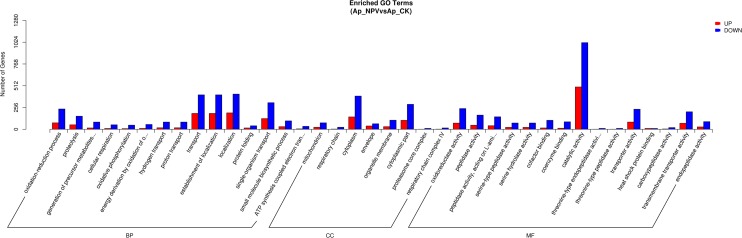
GO analysis of the DEGs. Genes with differential expression levels were classified as biological process, cellular component, and molecular function by WEGO according to the GO terms. The numbers of genes mapped to the GO terms are provided in the left panel.

The DEGs were assigned to various GO categories to determine their functional classifications (Fig 3). A total of 2,965 DEGs were classified into different GO categories, including 1,821 down-regulated and 1,144 up-regulated genes (S4 and S5 Tables). Overall, 8, 15 and 15 catalogs of cellular component, molecular function and biological process were clustered, respectively. In biological process, most DEGs were involved in transport, establishment of localization, localization and single-organism transport. In cellular component, the DEGs were enriched in cytoplasm, organelle membrane and cytoplasmic part. In molecular function, most GO terms were involved in enzyme activity, such as oxidoreductase activity, peptidase activity, serine hydrolase activity and catalytic activity, indicating that the enzyme system of *A*. *pernyi* midgut may be degenerated by ApNPV infection.

### KEGG pathway analysis of the differentially expressed genes

**Fig 4:**
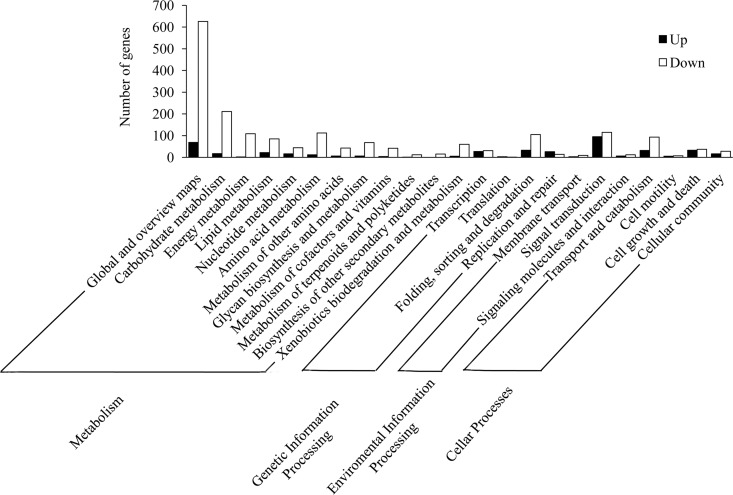
KEGG pathway analysis of the DEGs. The pathways were clustered into metabolism, genetic information processing, environmental information processing and cellular processes. The left panel lists the numbers of genes that mapped to each of the pathways.

**Fig 5:**
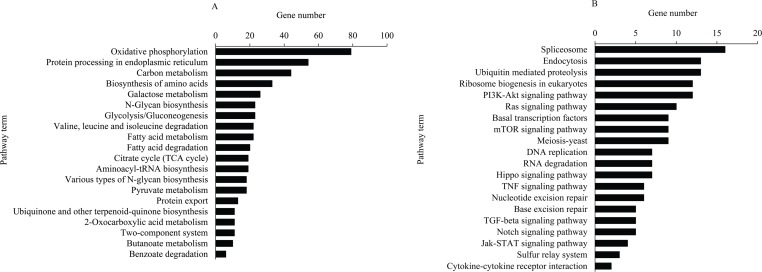
The top 20 pathways of the DEGs clustered by KEGG analysis. (A) The top 20 pathways for the down-regulated genes. (B) The top 20 pathways for the up-regulated genes.

A total of 911 DEGs, including 686 down-regulated and 225 up-regulated genes, were assigned to 155 KEGG pathways, including 114 pathways for the down-regulated genes (S6 Table) and 154 pathways for the up-regulated genes (S7 Table). The pathways were classified into the following four categories: metabolism, genetic information processing, environmental information processing and cellular processes (Fig 4). In the mapped pathways of the down-regulated genes, the abundant genes mapped onto protein processing in endoplasmic, oxidative phosphorylation, carbon metabolism and biosynthesis of amino acids. The up-regulated genes were mainly enriched in spliceosome, endocytosis, ubiquitin-mediated proteolysis and several signaling pathways (Fig 5).

### Validation of Illumina sequencing results by qRT-PCR

**Fig 6:**
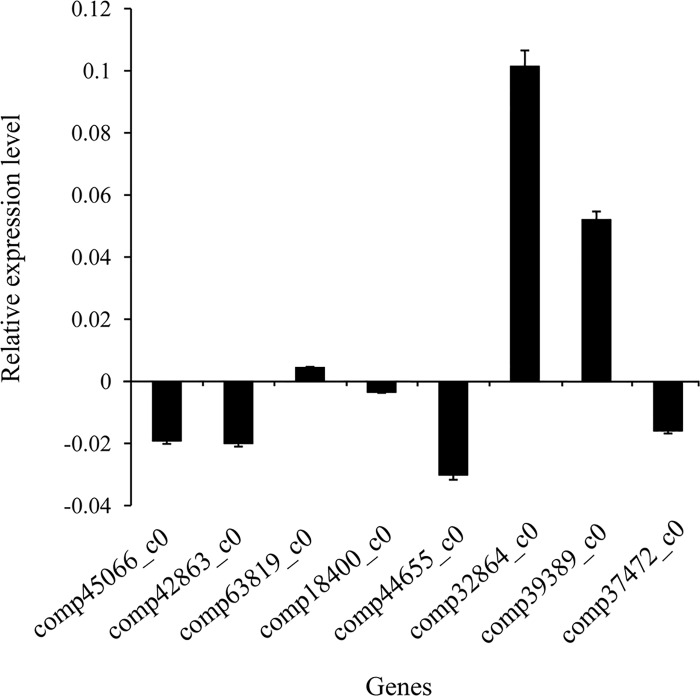
Quantitative RT-PCR analysis was used to validate the differentially expressed genes according to RNA-seq. X-axis represents the eight genes selected for qRT-PCR validation. comp45066_c0, heat shock protein 60 (*Chilo suppressalis*); comp42863_c0, heat shock protein 19.9 (*Bombyx mori*); comp63819_c0, apoptosis-inducing factor 3 (*Homo sapiens*); comp18400_c0, serine protease 3 (*Lonomia obliqua*); comp44655_c0, serine protease 5 (*Mamestra configurata*); comp32864_c0, serine protease inhibitor 12 (*Bombyx mori*); comp39389_c0, serine protease inhibitor 5 (*Bombyx mori*); comp37472_c0, cytochrome CYP324A1 (*Spodoptera littoralis*). Y-axis represents the relative expression level. The Ct values of each reaction were normalized to the endogenous control actin3.

To verify the RNA-seq results, we selected 8 DEGs, including 2 genes encoding HSPs, 1 gene encoding apoptosis-related protein, 2 genes encoding serine proteases, 2 genes encoding serpins and 1 gene encoding CYP, by designing specific primers for quantitative RT-PCR. The qRT-PCR results revealed different expression trends for the analyzed genes (Fig 6). Among the 8 DEGs, comp45066_c0, comp42863_c0, comp18400_c0, comp44655_c0 and comp37472_c0 displayed downward trends, whereas comp63819_c0, comp32864_c0 and comp39389_c0 exhibited rising trends. Besides, we also investigated the 25 top up-regulated and top down-regulated genes by qRT-PCR (S10 Table, S1 Fig). These results were consistent with the Illumina sequencing data.

## Discussion

*Antheraea pernyi* nucleopolyhedrovirus is an exclusive pathogen of *A*. *pernyi*. Like other NPVs, ApNPV also undergoes four temporally regulated transcription phases in the infection process: immediate early, early, late, and very late. The initial steps of viral infection mainly help the virus establish infection and selectively modulate certain categories of host genes. The movement of the nucleocapsid from the cytoplasm to the nucleus is essential for controlling host gene expression and producing viral progeny. The late transcription phase is focused mainly on DNA replication and structural gene expression [17, 18]. In this study, to ensure that the *A*. *pernyi* larvae acquired the NPV disease during the 3^rd^ instar, a high dose of polyhedra (4.05×10^6^ polyhedra/mL) was added to the meal to feed the *A*. *pernyi* larvae for three days. The time point at which polyhedra can be observed in the hemocytes in ApNPV-infected larva under the microscope was selected to compare the genes in the ApNPV-infected midguts that were differentially expressed relative to the controls. A total of 5,172 DEGs, including 2,183 up-regulated and 2,989 down-regulated genes, were identified through transcriptome sequencing. These DEGs identified during infection provide potential insights into the complex molecular mechanisms of the response to viral infection at the transcriptional level. Up-regulated genes may represent host cell responses to pathogens, whereas down-regulated genes may be partially attributed to the shut off of host macro molecular synthesis in favor of viral replication [19].

The midgut is the first line of innate immunity in insects and plays an important role in defending against pathogen invasion. In this study, several homologous genes possibly implicated in *A*. *pernyi*’s immune response against ApNPV infection were found by deep sequencing to be highly differentially expressed between the ApNPV-infected midguts and the controls. These genes are involved in several categories, including heat-shock protein, apoptosis, serpin, serine protease and cytochrome P450, as outlined in detail in Table 2. Together, these genes constitute a complex response to ApNPV infection in *A*. *pernyi* larvae.

**Table 2:** Homologous genes related to innate immunity of *A*. *pernyi*.

Description	Gene ID	Gene name	log_2_FC	Regulated	Species
Heat-shock protein	comp45066_c0	heat shock protein 60	-2.5198	down	*Chilo suppressalis*
	comp31442_c0	putative 10 kDa heat shock protein	-2.4893	down	*Danaus plexippus*
	comp44879_c0	heat shock protein cognate 3	-3.8206	down	*Papilio xuthus*
	comp35657_c0	heat shock protein 90 cognate	-3.8479	down	*Spodoptera litura*
	comp43361_c0	Hsp90-related protein TRAP1	-1.6533	down	*Bombyx mori*
	comp41370_c0	small heat shock protein 27.2	2.6588	up	*Spodoptera litura*
	comp41163_c0	heat shock factor-b	1.6748	up	*Bombyx mori*
	comp82669_c0	heat shock cognate 70 protein	-1.5983	down	*Danaus plexippus*
	comp18163_c0	small heat shock protein	2.239	up	*Danaus plexippus*
	comp41256_c0	19.5 kDa heat shock protein	2.0106	up	*Bombyx mori*
	comp46251_c0	small heat shock protein 22.2	0.94645	up	*Cydia pomonella*
	comp42863_c0	heat shock protein hsp 19.9	-2.4539	down	*Bombyx mori*
Apoptosis	comp18389_c0	inhibitor of apoptosis protein	-9.5849	down	*Bombyx mori*
	comp28678_c0	putative programmed cell death protein 8, mitochondrial precursor	1.9668	up	*Danaus plexippus*
	comp63819_c0	Apoptosis-inducing factor 3	1.4446	up	*Homo sapiens*
	comp19334_c1	caspase-1	1.4146	up	*Manduca sexta*
	comp42011_c0	putative apoptosis antagonizing transcription factor	1.1949	up	*Danausple*
	comp63250_c0	apoptosis inhibitor survivin	-1.1695	down	*Helicoverpa armigera*
	comp45307_c0	leucine-rich protein SCLP	3.994	up	*Manduca sexta*
Serine protease	comp43264_c4	serine protease 17	-3.9102	down	*Mamestra configurata*
	comp44654_c0	serine protease 36	-3.0685	down	*Mamestra configurata*
	comp29144_c0	trypsin-like serine protease	-3.5758	down	*Bombyx mori*
	comp43197_c0	chymotrypsin-like serine protease precursor	-2.6571	down	*Bombyx mori*
	comp44673_c0	serine protease 58	-2.516	down	*Mamestra configurata*
	comp18400_c0	serine protease 3	-2.3559	down	*Lonomia obliqua*
	comp33970_c0	serine protease	-3.3983	down	*Bombyx mandarina*
	comp44694_c0	serine protease 40	-2.1417	down	*Mamestra configurata*
	comp44605_c0	serine protease 11	-2.0678	down	*Mamestra configurata*
	comp39073_c0	trypsin-like serine protease 9	-2.0048	down	*Ostrinia nubilalis*
	comp44589_c0	serine protease 13	-1.8996	down	*Mamestra configurata*
	comp44655_c0	serine protease 5	-1.8303	down	*Mamestra configurata*
	comp39266_c0	serine protease 38	-1.7897	down	*Mamestra configurata*
	comp41147_c0	serine protease precursor	-3.2191	down	*Danaus plexippus*
	comp29660_c1	trypsin-like serine protease	3.0237	up	*Ostrinia nubilalis*
	comp39768_c0	putative trypsin-like serine protease	2.6678	up	*Danaus plexippus*
	comp26536_c0	serine protease precursor	-1.3874	down	*Bombyx mori*
	comp37774_c0	serine protease 2	-0.93433	down	*Mamestra configurata*
Serpin	comp32864_c0	serine protease inhibitor 12	2.8146	up	*Bombyx mori*
	comp42687_c0	serine protease inhibitor 100A	-1.4519	down	*Papilio polytes*
	comp60573_c0	serine protease inhibitor 4A	0.87984	up	*Bombyx mori*
	comp39389_c0	serine protease inhibitor 5	1.2074	up	*Bombyx mori*
Cytochrome P450	comp44198_c0	CYP6AB4	-2.5038	down	*Bombyx mandarina*
	comp40057_c0	cytochrome P450	-3.5675	down	*Bombyx mori*
	comp40128_c0	cytochrome P450 monooxygenase Cyp4M5	-1.7693	down	*Bombyx mori*
	comp93358_c0	cytochrome CYP4S8v1	3.6103	up	*Spodoptera littoralis*
	comp30550_c0	NADPH cytochrome P450 reductase	-1.2449	down	*Helicoverpa armigera*
	comp36738_c3	cytochrome P450	-3.5865	down	*Helicoverpa armigera*
	comp41253_c0	cytochrome P450	1.5251	up	*Antheraea pernyi*
	comp36738_c4	cytochrome P450 6AE8	-4.3915	down	*Bombyx mori*
	comp37472_c0	cytochrome CYP324A1	-1.7988	down	*Spodoptera littoralis*
	comp30405_c0	cytochrome P450 CYP304F2	-3.16	down	*Zygaena filipendulae*
	comp35531_c0	cytochrome P450 CYP9A22 precursor	1.7377	up	*Bombyx mori*
	comp43875_c0	cytochrome P450 CYP4L6 precursor	-3.0574	down	*Bombyx mori*

Heat-shock proteins (HSPs) are expressed constitutively in all cells and essential for several important cellular processes, such as protein folding and protecting proteins from denaturation or aggregation [20]. HSPs play an important role in antimicrobial and autoimmune responses and have potent effects in inducing antigen-specific immunities to bound materials upon immunization [21]. When insects are invaded by pathogens such as virus, HSPs function as molecular chaperones in preventing the accumulation of damaged proteins to maintain cellular homeostasis by refolding, stabilization, intracellular translocation and degradation of proteins. HSPs can also act as a ‘danger’-signaling molecule and send ‘danger signals’ to the immune system to generate a response to external stimuli, such as pathogens or environmental changes [21–23]. In this study, we identified 12 DEGs encoding different HSPs (Table 2), and the different expression trends of these genes indicate that some may be activated and others inhibited by ApNPV infection.

Apoptosis is the process of programmed cell death that is a normal component of the development of multicellular organisms. Apoptosis is required to destroy cells that may be infected with viruses or contain DNA damage that represent a threat to the integrity of the organism [24]. Apoptosis is one of the strategies by which antiviral defense mechanisms function in insects [25]. Some Lepidopteron insects resist baculovirus infection by sloughing off or selectively apoptosis the infected cells in the midgut epithelium [26]. We screened 7 DEGs involved in apoptosis from the obtained DEGs (Table 2). The up-regulation of the apoptosis-related genes (comp28678_c0, comp19334_c1 and comp45307_c0) and the down-regulation of the apoptosis inhibitors (comp18389_c0, comp42011_c0 and comp63250_c0) indicate that the host cells may initiate apoptosis to defend against the infection of ApNPV.

Serine proteases (SPs) are important enzymes that are not only involved in digestion, embryonic development and cell differentiation but also play important roles in resisting pathogen invasion [27]. SPs activate prophenoloxidase (PPO) and induce melanotic encapsulation of organisms by catalyzing PPO to active phenoloxidase (PO) and then initiate the innate immune response to pathogen invasion [28]. Serpins are serine protease inhibitors that exert tight regulation of proteolytic cascades important for many biological processes, such as the complement cascade, inflammation, and innate immunity in different organisms [29]. Serpins control the signals of innate immunity by regulating the activities of SPs to protect the host from pathogen infection [30]. By screening the DEGs, 18 SPs and 4 serpins were obtained (Table 2). We observed that the transcript level of comp39389_c0, encoding serine protease inhibitor 5 (Serpin5), in the ApNPV-infected midgut was higher than that in the control, consistent with the results of Bao *et al*. (2009) and Wu *et al*. (2011) [31, 32]. Serpin5 is a negative regulator of the Toll pathway and functions extracellularly, likely by blocking the proteolytic activation of Spaetzle, the Toll receptor ligand [33]. The differentially expressed SPs and serpins identified in this study indicate that they may play important roles in the immune response to ApNPV infection in the midgut of *A*. *pernyi*.

Cytochrome P450s (CYPs) are a superfamily of heme proteins that are involved in the metabolism a wide range of both endogenous and exogenous compounds [34]. In insects, CYPs are important for the detoxification of plant allelochemicals and insecticides [35, 36]. Twelve CYP DEGs were identified in the current study (Table 2), and the different expression levels of these genes imply that they may participate in the immune response induced by ApNPV infection.

The results of KEGG analysis showed that the down-regulated genes were mainly involved in several metabolic pathways such as oxidative phsophorylation, carbon metabolism, the biosynthesis of amino acids, galactose metabolism and fatty acid degradation (Fig 5A, S6 Table), suggesting that the functions of digestion and absorption of the *A*. *pernyi* midgut were degenerated due to ApNPV infection. Among the mainly pathways related to the up-regulated genes, several important immune pathways involved in defending against the virus infection were found (Fig 5B, S6 Table), including PI3K-Akt signaling pathway, Hippo signaling pathway, and JAK-stat signal pathway, indicating that the *A*. *pernyi* midgut initiated the immune system after the virus invasion. In conclusion, our results provide new clues for exploring the molecular mechanisms of ApNPV infection and the anti-ApNPV mechanisms of *A*. *pernyi*.

## Supporting Information

S1 Fig qRT-PCR results of the top 25 up-regulated and down-regulated genes. (DOCX)

S1 Table Primer pairs of candidate reference gene and target genes used for qRT-PCR analysis. (DOCX)

S2 Table The up-regulated genes in the ApNPV-infected *A*. *pernyi* midgut. (XLSX)

S3 Table The down-regulated genes in the ApNPV-infected *A*. *pernyi* midgut. (XLSX)

S4 Table GO annotations for the up-regulated genes. (XLSX)

S5 Table GO annotations for the down-regulated genes. (XLSX)

S6 Table KEGG pathways for the down-regulated genes. (XLSX)

S7 Table KEGG pathways for the up-regulated genes. (XLSX)

S8 Table GO annotations for all the differentially expressed genes. (XLSX)

S9 Table Clusters of orthologous group classifications of the unigenes. (XLSX)

S10 Table The top 25 up-regulated and down-regulated genes. (DOCX)
