# Supplementary material for: Transcriptome Analysis of the Midgut of the Chinese Oak Silkworm Antheraea pernyi Infected with Antheraea pernyi Nucleopolyhedrovirus
Source: PLoS One. 2016 Nov 7;11(11):e0165959. doi: 10.1371/journal.pone.0165959 (PMC5098726; doi:10.1371/journal.pone.0165959)
Supplement: S1 Fig — (DOCX) [file pone.0165959.s001.docx]

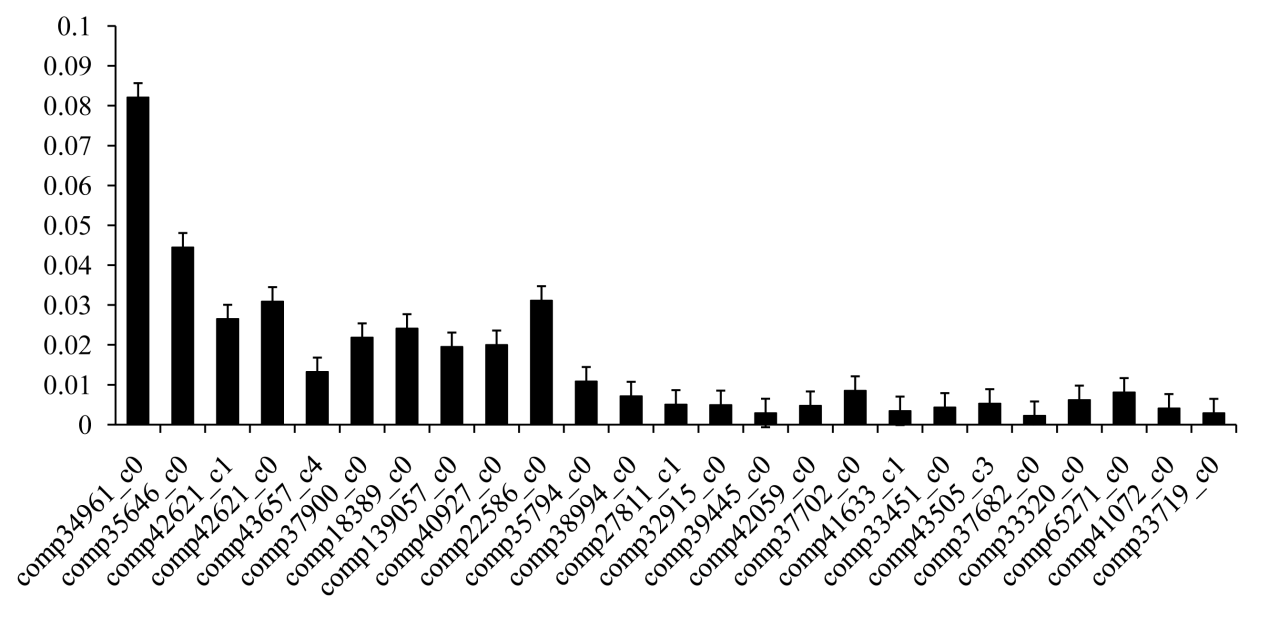


A


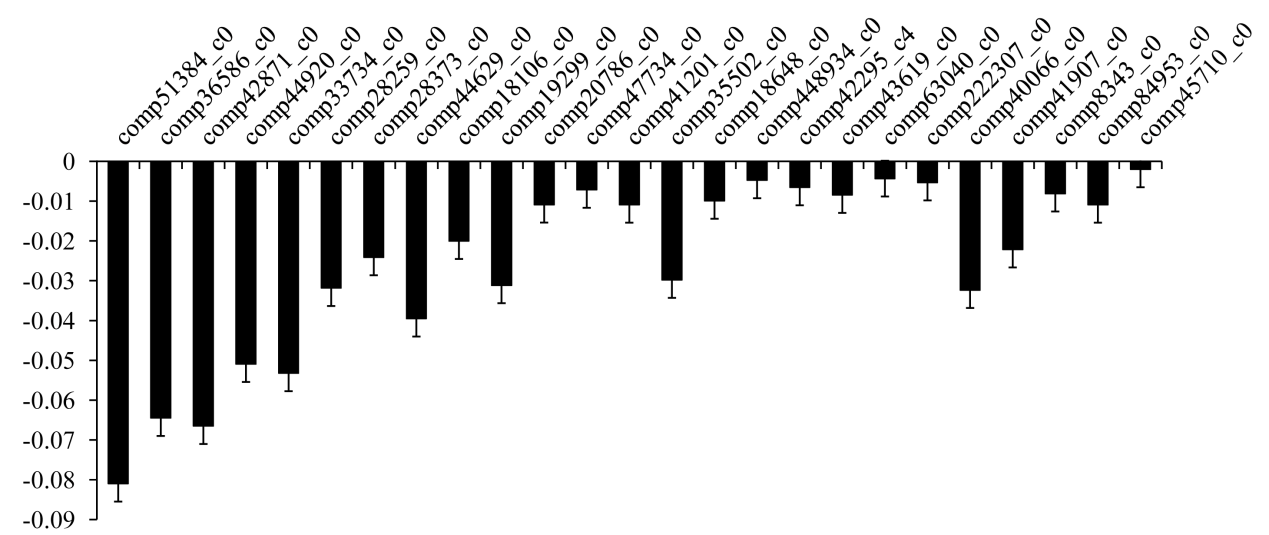


B

**S1 Fig. qRT-PCR results of the top 25 up-regulated (A) and down-regulated (B) genes.** Bscissa represents Gene ID. Vertical coordinate represents the relative expression level. The Ct values of each reaction were normalized to the endogenous control actin3.
