# Supplementary material for: Transcriptome Analysis of the Midgut of the Chinese Oak Silkworm Antheraea pernyi Infected with Antheraea pernyi Nucleopolyhedrovirus
Source: PLoS One. 2016 Nov 7;11(11):e0165959. doi: 10.1371/journal.pone.0165959 (PMC5098726; doi:10.1371/journal.pone.0165959)
Supplement: S1 Table — (DOCX) [file pone.0165959.s002.docx]

**S1 Table. Primer pairs of candidate reference genes and target genes used for qRT-PCR analysis.**

| **Gene ID** | **Gene name** | **Primer sequence (5’-3’)** |
| --- | --- | --- |
| comp45066_c0 | heat shock protein 60 (*Chilo suppressalis*) | F: GTGCCAAGGTCGAGTTCCAG  R: CAATAGCCATGTCACTGAGAG |
| comp42863_c0 | heat shock protein 19.9 (*Bombyx mori*) | F: GTCCACTAGTCAACAACG  R: GAACTGCCGCGATATGTAC |
| comp18400_c0 | serine protease 3 (*Lonomia obliqua*); | F: CAGCTCAGGAGGGCCAGTTC  R: GTCGCTGATGTCGTGCGGC |
| comp44655_c0 | serine protease 5 (*Mamestra configurata*) | F: ACTACCCCTACATGTCCAAT  R: ACGATCGCAACGTCATTGTC |
| comp37472_c0 | cytochrome CYP324A1 (*Spodoptera littoralis*) | F: GATCCTCTCTGGTCTTCAC  R: ATACCAAATGCAGCCTCA |
| comp63819_c0 | apoptosis-inducing factor 3 (*Homo sapiens*) | F: TGCAAGTGAGATCTCAGGA  R: TCAAAGTCTCTCACTGTGA |
| comp32864_c0 | serine protease inhibitor 12 (*Bombyx mori*) | F: TTGCTACAGAGCCACCTGT  R: CCTCAACTTCCTGTTCCTG |
| comp39389_c0 | serine protease inhibitor 5 (*Bombyx mori*) | F: CAATGACTTCACCATTTAT  R: TGTCAAAATCATCCTAGAT |
|  | actin3 | F: ACCAACTGGGACGACATGGAG  R: TCTCTCTGTTGGCCTTTGGGT |
