# Supplementary material for: Transcriptome Analysis of the Midgut of the Chinese Oak Silkworm Antheraea pernyi Infected with Antheraea pernyi Nucleopolyhedrovirus
Source: PLoS One. 2016 Nov 7;11(11):e0165959. doi: 10.1371/journal.pone.0165959 (PMC5098726; doi:10.1371/journal.pone.0165959)
Supplement: S10 Table — (DOCX) [file pone.0165959.s011.docx]

**S10 Table. The top 25 up-regulated and down-regulated genes.**

**A** **The top 25 up-regulated genes**

| **Gene ID** | **Gene name** |
| --- | --- |
| comp34961_c0 | Transcription termination factor 2 |
| comp35646_c0 | superoxide dismutase |
| comp42621_c1 | Late expression factor 4 |
| comp42621_c0 | chitinase 7 |
| comp43657_c4 | enhancing factor precursor |
| comp37900_c0 | protein tyrosine phosphatase |
| comp18389_c0 | inhibitor of apoptosis protein |
| comp139057_c0 | asteroid homolog 1 |
| comp40927_c0 | regulatory-associated protein of TOR |
| comp22586_c0 | Gag protein |
| comp35794_c0 | Fatty acid-binding protein 2 |
| comp38994_c0 | fatty acid synthase-like isoform 2 |
| comp27811_c1 | hormone receptor 4 |
| comp32915_c0 | trypsin-like proteinase |
| comp39445_c0 | mitochondrial phosphoenolpyruvate carboxykinase isoform 1 |
| comp42059_c0 | endonuclease-reverse transcriptase |
| comp37702_c0 | oxidase |
| comp41633_c1 | potassium-dependent sodium-calcium exchanger |
| comp33451_c0 | chitin binding peritrophin-A |
| comp43505_c3 | transcription factor SOX-14 |
| comp37682_c0 | putative fatty acid synthase |
| comp33320_c0 | phytanoyl-CoA dioxygenase peroxisomal precursor |
| comp65271_c0 | acidic lipase |
| comp41072_c0 | Zinc finger protein 294 |
| comp33719_c0 | karyopherin alpha 3 |

**B** **The top 25 down-regulated genes**

| **Gene ID** | **Gene name** |
| --- | --- |
| comp51384_c0 | cuticular protein RR-1 |
| comp36586_c0 | transferrin precursor |
| comp42871_c0 | luciferase |
| comp44920_c0 | alpha amylase precursor |
| comp33734_c0 | lactase-phlorizin hydrolase |
| comp28259_c0 | putative alcohol dehydrogenase |
| comp28373_c0 | beta-glucosidase precursor |
| comp44629_c0 | trypsin |
| comp18106_c0 | hydroxybutyrate dehydrogenase |
| comp19299_c0 | 3-dehydroecdysone 3 alpha-reductase |
| comp20786_c0 | alcohol dehydrogenase |
| comp47734_c0 | juvenile hormone acid methyltransferase |
| comp41201_c0 | AMP dependent coa ligase |
| comp35502_c0 | carbonyl reductase |
| comp18648_c0 | apolipophorin III |
| comp448934_c0 | apolipophorin precursor protein |
| comp42295_c4 | ecdysteroid 22-kinase |
| comp43619_c0 | putative carbonic anhydrase |
| comp63040_c0 | alkaline phosphatase |
| comp222307_c0 | sugar transporter |
| comp40066_c0 | ATP-binding cassette transporter |
| comp41907_c0 | kynurenine 3-monooxygenase |
| comp8343_c0 | chondroitin 4-sulfotransferase |
| comp84953_c0 | aldo-keto reductase |
| comp45710_c0 | beta-glucosidase precursor |
